# Conserved post-odor dynamics in the olfactory systems of mice and locusts

**DOI:** 10.1016/j.isci.2026.115373

**Published:** 2026-03-16

**Authors:** Elizabeth H. Moss, Doris Ling, Cameron L. Smith, Feiyang Deng, Ryan Kroeger, Jacob Reimer, Baranidharan Raman, Benjamin R. Arenkiel

**Affiliations:** 1 Department of Molecular and Human Genetics, Baylor College of Medicine, Houston, TX 77030, USA; 2 Department of Biomedical Engineering, Washington University in St. Louis, St. Louis, MO 63105, USA; 3 Department of Neuroscience, Baylor College of Medicine, Houston, TX 77030, USA

**Keywords:** biological sciences, neuroscience, sensory neuroscience

## Abstract

Interpreting chemical information and translating it into ethologically relevant output is a shared challenge of olfactory systems across species, but are olfactory computations conserved across species to overcome these common challenges? To investigate this, we compared neural activity in the locust antennal lobe (AL) and mouse olfactory bulb (OB) both during and after odor presentations. We found that odors activated nearly mutually exclusive neural ensembles during odor presentations (“ON response”) and after the termination of the odor stimulus (“OFF response”). ON and OFF responses evoked by a single odor were anticorrelated with each other. Inverted OFF responses persisted long after odor termination in both AL and OB, and enhanced contrast between odors that were experienced close together in time. Together our results show how post-odor neural activity, relative to odor-evoked activity, is similar across two distinct species, revealing a conserved mechanism for enhancing contrast between odors at the neural level.

## Highlights

• Odor offset elicits anticorrelated activity in mouse and locust olfactory circuits
• OFF responses form persistent, inverted neural representations of recent odors
• Post-odor activity enhances contrast between closely spaced odor encounters
• Odors remain decodable from population activity for several seconds after odor offset

## Graphical abstract

**Figure:**
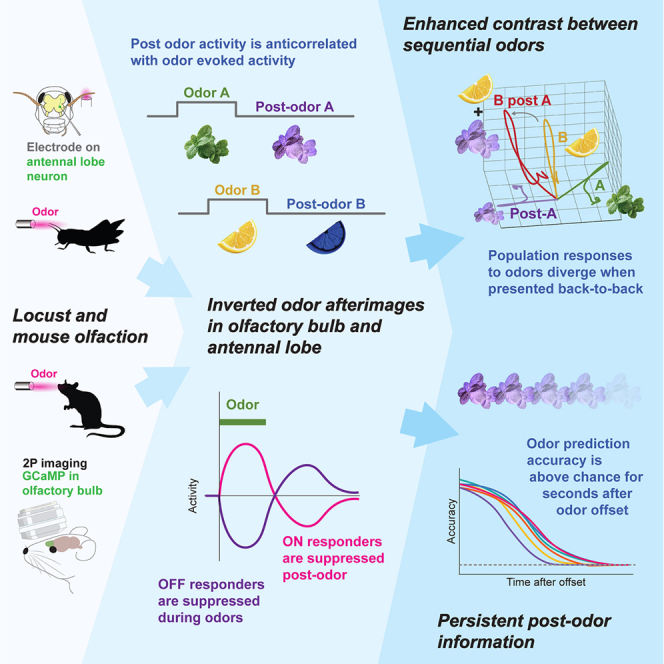

## Introduction

Olfactory systems across species have the common requirement of translating chemical information into neuronal activity to drive behaviors. A key aspect of this transformation is the temporal structure of odor-evoked activity, which includes not only responses during odor presentations, but also odor-specific neural activity that persists after an odor stimulus ends. Although vertebrate and invertebrate olfactory systems and odor-guided behaviors differ markedly, both must process temporally structured information to extract behaviorally relevant information.

While insect olfaction relies on odor molecules to make direct contact with external antennae, mammalian olfaction requires respiration to internalize odor molecules and bring them into contact with the olfactory epithelium.^1^ While both vertebrate and invertebrate systems employ large families of seven-transmembrane proteins for sensing odors, in vertebrates they function as G-protein coupled receptors,^2^ while in insects they act as ligand-gated ion channels.^3^ These differences in the structure of sensory input generate different temporal dynamics in odor inputs and odor-evoked neuronal activity.^4^^,^^5^^,^^6^ Downstream of sensory neuron input, early olfactory circuits of the invertebrate antennal lobe (AL) have been studied predominantly as feedforward neural networks.^7^^,^^8^^,^^9^ In comparison, their vertebrate counterparts in the olfactory bulb (OB) receive massive centrifugal feedback projections,^10^^,^^11^^,^^12^^,^^13^ suggesting that complex processing begins at the first circuit node that receives chemosensory input. Despite these differences in sampling methods and circuit architecture, olfactory systems have shared common needs across species. These include sensitivity and specificity across a complex chemical space, a broad dynamic range, the ability to filter and emphasize different inputs, and the capacity to distinguish distinct odors from complex backgrounds.^14^^,^^15^^,^^16^^,^^17^^,^^18^^,^^19^^,^^20^^,^^21^

Computations like background subtraction and contrast enhancement, in particular, may rely on neural activity that persists after odor offset (OFF responses), providing a temporal context against which subsequent odors can be compared. Stimulus OFF responses are common across species and sensory circuits, and responses to stimuli often continue well after stimulus termination as sensory “aftereffects”.^22^^,^^23^^,^^24^^,^^25^ However, the computational purpose of a stimulus-specific “afterimage” in olfactory processing remains unknown. In particular, it is unknown whether OFF responses preserve or transform odor relationships, and whether they can provide a useable substrate for computations like contrast enhancement, background subtraction, temporal segmentation of odor encounters, or short-term olfactory memory. Moreover, it is unclear whether the structure of OFF response dynamics differs across species to reflect species-specific circuit motifs, or whether convergent dynamics can arise from different receptor repertoires and/or neural architectures. These considerations raise a central question: do olfactory systems with fundamentally different inputs and circuit architectures converge on analogous mechanisms to carry out similar, simple computations?

To address this question, we compared population-level odor response dynamics across insects and mammals, with an emphasis on post-odor activity. Using *in vivo* electrophysiology in the locust AL, and mesoscale two-photon calcium imaging of the mouse OB, we captured neuronal ensemble responses to passive odor presentations. These complementary approaches provided the unique advantage of capturing odor response dynamics with different levels of spatial and temporal resolution. Despite the different resolutions of these methods and the different cellular sources of odor response signals (pools of dendrites in mice vs. single neurons in locust), we found notable similarities in the patterns of odor response dynamics between mice and locusts.

In both mice and locusts, we found that ensembles of neurons activated during odor presentations (ON responses) are different from those that become responsive after stimulus termination, resulting in nearly mutually exclusive ON and OFF neuronal response patterns across odors. Strikingly, we found that neuronal ensembles in both locust AL and mouse OB show responses to the onset and the offset of individual odors that are inverted relative to each other (i.e., ON and OFF population response vectors are anticorrelated). We propose that such similar neural dynamics across mouse and locust provide a neural underpinning of two emergent computations shared by both species: contrast enhancement and short-term olfactory memory. Such computations may be considered critical for animals navigating the world, following odor trails, encountering new odors within complex backgrounds, and sensing odors in temporally complex plumes. Together, our results suggest that these fundamental building blocks of olfactory computations are conserved across insect and mammalian olfactory systems, revealing common and potentially indispensable mechanisms of olfactory processing.

## Results

### OFF responses form anticorrelated odor afterimages across species

**Figure 1:**
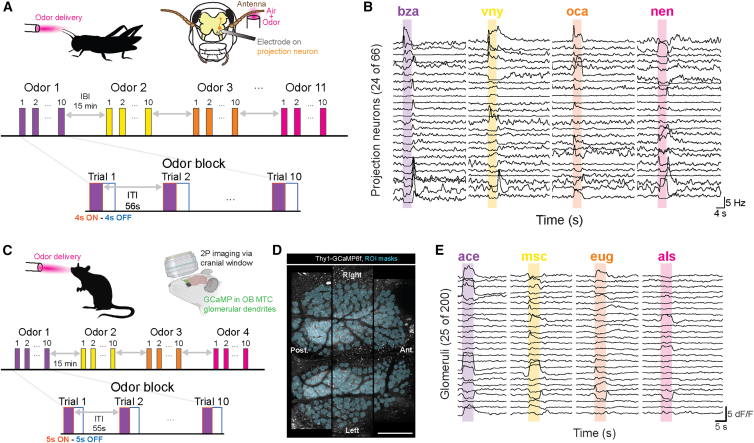
Odor ON and OFF responses recorded from locust antenna lobe and mouse olfactory bulb (A) Schematic of the experimental design. (top) Multi-unit extracellular electrophysiological signals were recorded from the projection neurons (PN) in the locust antennal lobe (AL). (bottom) Odors were presented to locusts in blocks of ten trials. A 15-min gap separated blocks of different odors. (B) Representative trial-averaged PSTH traces of 24 PNs in response to benzaldehyde (bza), 4-vinyl anisole (vny), octanol (oct), and z-3-nonen-1-ol (nen). (C) Schematic of the experimental design. (top) Odor-evoked activity was observed from mitral and tufted cell (MTC) glomerular dendrites with mesoscale two-photon calcium imaging through chronic cranial windows over the mouse OB. (bottom) Odors were presented for 5 s, separated by 55 s intertrial intervals, in blocks of ten trials. 15-min periods of no odor stimulation separated blocks of different odors. (D) Representative trial-averaged image of GCaMP fluorescence in the olfactory bulb of a Thy1-GCaMP6f mouse with ROIs overlaid on glomeruli from bilateral olfactory bulbs. Scale bars, 500 μm. (E) Fluorescence traces (dF/F) from 25 representative glomeruli showing responses to acetophenone (ace, purple), methyl salicylate (msc, yellow), eugenol (eug, orange), and allyl sulfide (als, pink).

To compare how early olfactory circuits in insects and mammals represent odor dynamics over time, we recorded population-level activity in the locust AL and mouse OB during passive odor presentations (Table 1). In locusts, we used extracellular electrophysiology to record spiking activity from projection neurons (PNs) in the AL (Figure 1A). This method provided high temporal resolution and access to single-neuron responses, allowing us to capture precise patterns of activity across PN ensembles throughout odor presentation and after stimulus offset (Figure 1B). In mice, we used mesoscale two-photon calcium imaging^26^ to measure glomerular responses from mitral and tufted cell dendrites in Thy1-GCaMP6f (GP5.11, JAX: 024339) animals (Figure 1C). This imaging approach captured odor-evoked activity from hundreds of glomeruli (median: 215 per session) across the dorsal surface of bilateral OBs (Figure 1D) with cellular spatial resolution and temporal resolution limited primarily by GCaMP response kinetics (Figure 1E). Together, these complementary techniques enabled species-appropriate, high-resolution recordings of odor response dynamics at the first stage of central olfactory processing.

**Table 1:** Summary of datasets used in each figure

Figure	Dataset	Stimulus presentations analyzed
Figure 1B	locust dataset 1: 66 PNs across 15 animals	11 odors, solitary block structure
Figures 1D and 1E	mouse 2, session 3: 198 glomeruli	4 odors, solitary block structure
Figures 2A–2E	locust dataset 1: 66 PNs across 15 animals	11 odors, solitary block structure
Figures 2F–2I	mouse 2, session 3: 198 glomeruli	4 odors, solitary block structure
Figure 2J	7 mouse datasets: m1 session 1, 73 glomeruli; m2 session 1, 210 glomeruli; m2 session 2, 153 glomeruli; m2 session 3, 198 glomeruli; m3 session 1, 286 glomeruli; m4 session 1, 219 glomeruli; m4 session 2, 318 glomeruli	4 odors, solitary block structure
Figures 3A–3D	locust dataset 1: 66 PNs across 15 animals	11 odors, solitary block structure
Figures 3E–3H	mouse 2, session 3: 198 glomeruli	4 odors, solitary block structure
Figures 3G–3H	7 mouse datasets: m1 session 1, 73 glomeruli; m2 session 1, 210 glomeruli; m2 session 2, 153 glomeruli; m2 session 3, 198 glomeruli; m3 session 1, 286 glomeruli; m4 session 1, 219 glomeruli; m4 session 2, 318 glomeruli	4 odors, solitary block structure
Figures 4B–4E	reanalysis of locust dataset from^27^: 85 PNs across 9 animals	6 odors, sequential block structure
Figures 4G and H	mouse 4, session 1: 219 glomeruli	4 odors, sequential block structure
Figures 4I and 4J left	3 mouse datasets: m3 session 1, 286 glomeruli; m4 session 1, 219 glomeruli; m4 session 2, 318 glomeruli	4 odors, sequential block structure
Figures 4J right	4 mouse datasets: m1 session 1, 73 glomeruli; m2 session 1, 210 glomeruli; m2 session 2, 153 glomeruli; m2 session 3, 198 glomeruli	4 odors, sequential block structure
Figure 5	reanalysis of locust dataset from^27^: 85 PNs across 9 animals	7 odors, solitary block structure
Figures 6A–6D	mouse 4, session 2: 318 glomeruli	4 odors, solitary block structure
Figures 6E and 6F	6 mouse datasets: m2 session 1, 210 glomeruli; m2 session 2, 153 glomeruli; m2 session 3, 198 glomeruli; m3 session 1, 286 glomeruli; m4 session 1, 219 glomeruli; m4 session 2, 318 glomeruli	4 odors, solitary block structure
Figures 6G and 6H	mouse 9, session 1; 286 glomeruli	11 odors, random structure
Figures 6I	8 mouse datasets: m5 session 1 175 glomeruli; m6 session 1, 340 glomeruli; m7 session 1, 323 glomeruli; m8 session 1, 306 glomeruli; m9 session 1, 286 glomeruli; m9 session 2, 204 glomeruli; m9 session 3, 250 glomeruli; m10 session 1, 280 glomeruli	11 odors, random structure

Locust and mouse datasets used for analyses and figure generation are listed for each figure along with their characteristics.

**Figure 2:**
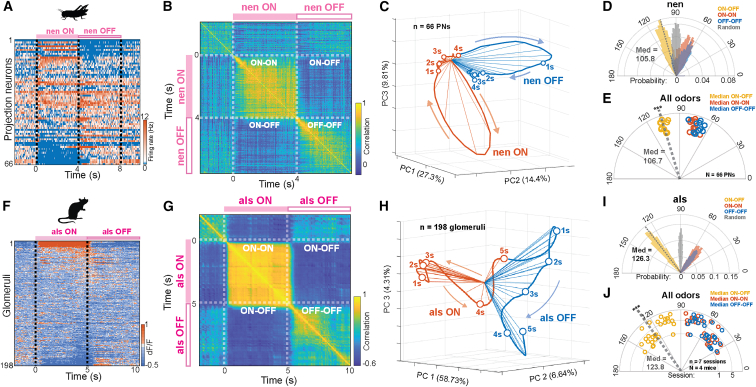
Odor ON and OFF responses are anticorrelated (A) Trial-averaged responses of 66 PNs to z-3-nonen-1-ol (nen) sorted by ratio of ON to OFF response. (B) Representative temporal correlation of trial-averaged PN activity. (C) Representative PCA trajectories (thick lines) of trial-averaged PN activity during the 4-s ON period of nen exposure (orange) and 4-s OFF period (blue). Spokes (thin lines) represent 200 ms time intervals and open circles mark 1 s intervals. (D) Distribution of angular separation between PN population vectors for a representative odor (nen). Angular separations are calculated between any ON response vector with other ON response vectors (orange), any OFF response vector with any other OFF response vector (blue), or any ON response vector with any OFF response vector (yellow). Distribution of angular separation between random vectors (gray) are shown as a control. Median values of ON vs. OFF angular separation distributions indicated with dashed lines and gray text. (E) Summary of the median angular separations for all 11 odors showing ON vs. ON period median angles (orange, median = 75.2 ± 3.9° SD), OFF vs. OFF period median angles (blue, median = 70.6 ± 4.7° SD) and ON vs. OFF period median angles (yellow, median = 106.7 ± 2.9° SD, ∗∗∗ one-sided *p* = 4.9 × 10^−4^, Wilcoxon signed-rank test vs. 90°). Points corresponding to different odors are offset along the radius for visualization. Mean ON vs. OFF angular distance across odors indicated by the dashed gray line. (F) Heatmap showing trial-averaged dF/F traces from all glomeruli in one mouse in response to allyl sulfide (als ON, filled pink bar) and after odor offset (als OFF, open pink bar). (G) Representative correlation matrix summarizing the similarity between trial-averaged glomerular responses evoked by allyl sulfide (als, pink bar) during ON and OFF time periods. (H) PCA trajectories of trial averaged glomerular population response during allyl sulfide presentation (als ON, orange) and after allyl sulfide presentation (als OFF, blue). (I) Distribution of angular distance between pairs of glomerular response vectors for a representative odor (aLs). Angular separations are calculated between vectors within the ON (orange) and OFF (blue) periods and between any ON response vector and any OFF response vector (yellow). Distribution of angular separation between random vectors (gray) are shown as a control. Median values of ON vs. OFF angular separation distributions indicated with dashed lines and gray text. (J) Summary of the median angular distances for 4 odors (*n* = 7 imaging sessions from 4 mice) showing ON vs. ON period median angles (orange, median = 56.5 ± 16.7° SD), OFF vs. OFF period median angles (blue, median = 56.2 ± 16.9° SD) and ON vs. OFF period median angles (yellow, median = 123.8 ± 15.8° SD, ∗∗∗ one-sided *p* = 7.5 × 10^−9^, Wilcoxon signed-rank test vs. 90°). The mean of the ON vs. OFF angles is shown as the dashed gray line and text. Radii indicate different imaging sessions (*n* = 7 imaging sessions from = 4 mice).

Notably, the majority of locust AL PNs that were responsive during odor presentations became silent after stimulus termination. Conversely, neurons that were suppressed during odor presentation became activated after stimulus termination (Figure 2A). To quantitatively examine the dissimilarity between ensemble ON and OFF responses, we applied a correlation analysis using high-dimensional response vectors. For this we calculated pairwise correlations between all PN response vectors observed at different time points as a correlation matrix (Figure 2B). Each row or column of the correlation matrix indicates the similarity between an ensemble response vector at a specific time point with all other PN response vectors observed over time. Neural responses over time within the ON period (for the duration of the odor presentation) were highly correlated among one another (ON-ON). Similarly, OFF responses were highly correlated with one another (OFF-OFF). However, the cross-correlation between any ON response time point with any OFF response time point (ON-OFF) was greatly reduced compared to intra-period correlations. These results show that ON and OFF neural response neural responses were quantitatively distinct from one another.

To qualitatively visualize distinct odor ON and OFF dynamics, and monitor how they evolved over time, we used a trajectory analysis. For this, we evaluated spike counts from 50 ms time bins across all recorded neurons as a high dimensional vector. These vectors were projected onto three dimensions that captured a large fraction of the variance (Figure 2C; Principal-component analysis or PCA; *n* = 66 PNs; ∼50% variance captured). Neural response vectors during ON periods (orange trajectories; 4 s during the odor presentation) were distinct from response vectors during OFF periods (blue trajectories; 4 s from the termination of odorant) for the duration of the 4 s OFF response window. Dimensionality reduced vectors were used for visualization only. All analyses were carried out on full-dimensional data.

Consistent with the observed anticorrelation between ON and OFF responses, we found that the angles between ON and OFF response vectors (a measure of dissimilarity) were consistently greater than 90° across a panel of odors (Figure 2D). For each odor, we calculated the angle between full-dimensional PN response vectors for time points during the odor presentation and after the odor presentation. Vector comparisons fell into three groups: (1) comparing vectors from the ON period to other vectors from the ON period (ON vs. ON; 80 vectors, 3160 pairwise comparisons), (2) comparing vectors from the ON period to vectors from the OFF period (80 ON vs. 80 OFF vectors; 6400 pairwise comparisons), and (3) comparing vectors from the OFF period to other vectors from the OFF period (OFF vs. OFF; 80 vectors, 3160 pairwise comparisons). In contrast, angles between random vectors of the same dimensionality clustered with means and medians around 90° (gray distributions). To perform quantitative comparisons across odors, we computed the median angular separation for each odor. Notably, for all odors the median angle between the ON and OFF responses was more than 90° (median = 106.8° ± 2.9° SD, *n* = 11 odors, one-sided *p* = 4.9 × 10^−4^, Wilcoxon signed-rank test vs. 90°), reflecting the negative correlation between ON and OFF responses (Figure 2E). These data are consistent with our prior results,^27^ and generalize our previous findings by showing that distinct neural ensembles are activated during and after presentations of a wide panel of odors.

We found that, like locust PNs, distinct sub-populations of mitral and tufted cells uniquely responded to odor onsets and offsets (Figure 2F). These data are consistent with previous studies reporting that neural responses in the mouse OB after termination of a stimulus are distinct from activity during stimulus presentations.^23^ However, similar to locust PNs, the pairwise correlation of glomerular ON and OFF response vectors revealed that odor ON and OFF responses were not just distinct but were anticorrelated (Figure 2G). Dimensionality reduction by principal components showed that the temporal trajectories of both ON and OFF responses to single odor presentations were distinct (Figures 2H and 2I). ON-OFF population angles were strongly biased above orthogonality (median 123.8° ± 15.8° SD, *n* = 28). This difference from 90° was highly significant (one-sided *p* = 7.5 × 10^−9^, Wilcoxon signed-rank test vs. 90°, Figure 2J), revealing that, as in the locust, odor ON and OFF responses in mouse OB glomeruli are inverted relative to each other. Together these data suggest that odor-evoked aftereffects in the mouse OB and locust AL form an inverted odor afterimage. Notably, the anticorrelation between ON and OFF responses imaged from dendrites in the mouse OB was larger than that observed from locust PN recordings. This is unlikely due to differences in the temporal dynamics between recording modalities given that, (1) we would expect to observe sharper transitions from ON to OFF and a stronger anticorrelation using the method with higher temporal resolution, and (2) in locust experiments, action potentials are pooled into 50 ms bins to calculate firing rates, making the temporal resolutions of the two recoding modalities comparable. What seems more likely is that the stronger anticorrelation effects observed in the mouse OB from imaging populations of glomeruli simultaneously in individual trials may result from pooling dendritic responses from correlated sister cells innervating the same glomeruli, thus reducing some neuron-to-neuron and trial to trial variability.

### ON and OFF responses occupy distinct regions of feature space

**Figure 3:**
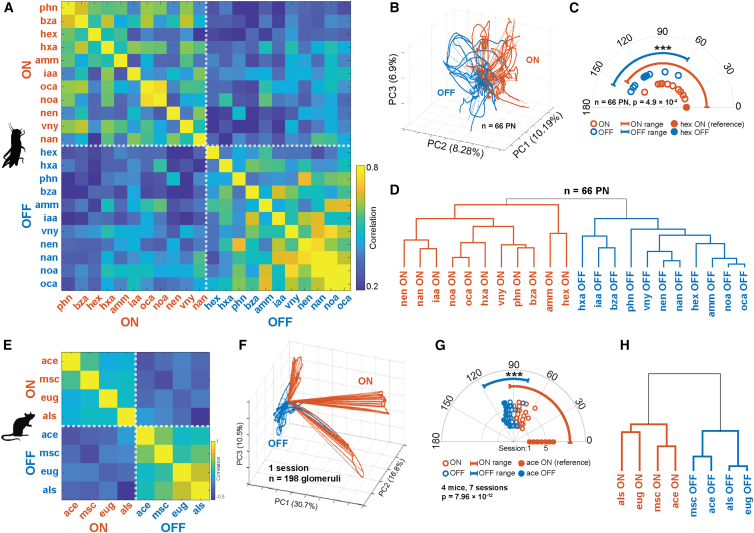
ON and OFF responses contain distinct odor specific information (A) Correlation of trial-averaged locust AL PN ON and OFF responses to all eleven odorants in the panel. (B) PCA trajectories of odor-evoked ON (orange) and OFF (blue) responses to all 11 odors in the panel. (C) Polar plot of angular distances between the hex ON response vector (reference, solid orange circle; averaged over 4 s odor presentation time window) and the ON response vectors of the 10 other odors (orange open circles, median = 59.5° ± 43.5° SD). Angular distances between the hex ON response vector and the OFF responses of all odors (blue open circles, median = 118.1° ± 24.5° SD) including hex OFF response (solid blue circle) are shown. Arcs (thick lines) show the range of angular distances observed for ON-ON angular distances and compares the same with ON-OFF angular distances. ∗∗∗ one-sided *p* = 4.9 × 10^−4^, Mann-Whitney U test comparing ON and OFF vs. reference angles. (D) Hierarchical clustering of ensemble locust AL PN ON and OFF responses. (E) Correlation matrix summarizing the similarity between glomerular ON and OFF responses evoked by four odors. (F) PCA trajectories of odor-evoked ON (orange) and OFF (blue) responses to four odors: ace, msc, eug, and als. (G) Polar plot showing the angular distance between ace ON response vector (reference, solid orange circles), ON response vectors of the 3 other odors (open orange circles, median = 44.7° ± 37.9° SD), ace OFF response vector (solid blue circle), or OFF response vector of the three other odors (open blue circles, median = 96.7° ± 9.0° SD. Radii indicate responses from different imaging sessions (*n* = 7 imaging sessions from 4 mice). Arced lines show the range of angular distances between ON responses (orange). ∗∗∗ one-sided *p* = 7.96 × 10^−12^, Mann-Whitney U test comparing ON and OFF vs. reference angles. (H) Hierarchical clustering of glomerular ON and OFF response vectors from a representative imaging session.

To evaluate the separability of population odor responses by odor identity during ON and OFF time periods we next calculated the correlations between ON and OFF responses of all odors for mice and locusts. In locusts the intra-period correlations (ON vs. ON and OFF vs. OFF) were relatively high, while inter-period correlations (ON vs. OFF) were comparatively low (Figure 3A). PCA using neural responses during both ON and OFF periods revealed that across odors, ON and OFF responses in the locust AL occupied different regions of PCA space (Figure 3B). To quantitatively validate the observations from PCA analysis, we used high-dimensional response vectors to calculate angular separation between the ON response elicited by a reference odor alongside the ON and OFF responses elicited by all odors in the odor panel. Separations between the ON response of the reference odor and the ON responses to all other odors were significantly smaller than the those observed between the ON response for the reference odor and the OFF response to all odors (one-sided *p* = 4.9 × 10^−4^, Mann–Whitney U test) indicating that, as a group, OFF responses are more dissimilar to ON responses than ON responses are to each other (Figure 3C). Hierarchical clustering of high dimensional ON and OFF responses also revealed that PN responses during ON and OFF periods formed distinct response clusters (Figure 3D). Together, these data suggest that ON responses for different odors were markedly more similar to each other than they were to OFF responses across odors in the locust AL. Consistent with the locust results, correlating glomerular response vectors across ON and OFF periods of different odor presentations in mice demonstrated that ON responses were distinct from OFF responses in general (Figure 3E). ON responses in mice occupied a distinct region of PCA space compared to OFF responses (Figure 3F), and the angular separations between a reference odor and all other odor ON responses were significantly smaller than those between the reference ON response and all odor OFF responses (one sided *p* = 7.96 × 10^−12^, Mann-Whitney U test, Figure 3G). Moreover, hierarchical clustering of mouse odor responses cleanly separated ON and OFF responses (Figure 3H), showing that, as in the locust AL, ON, and OFF responses in the mouse OB represent distinct patterns of neuronal activity.

### History-dependent suppression is enhanced between sequentially presented odors

**Figure 4:**
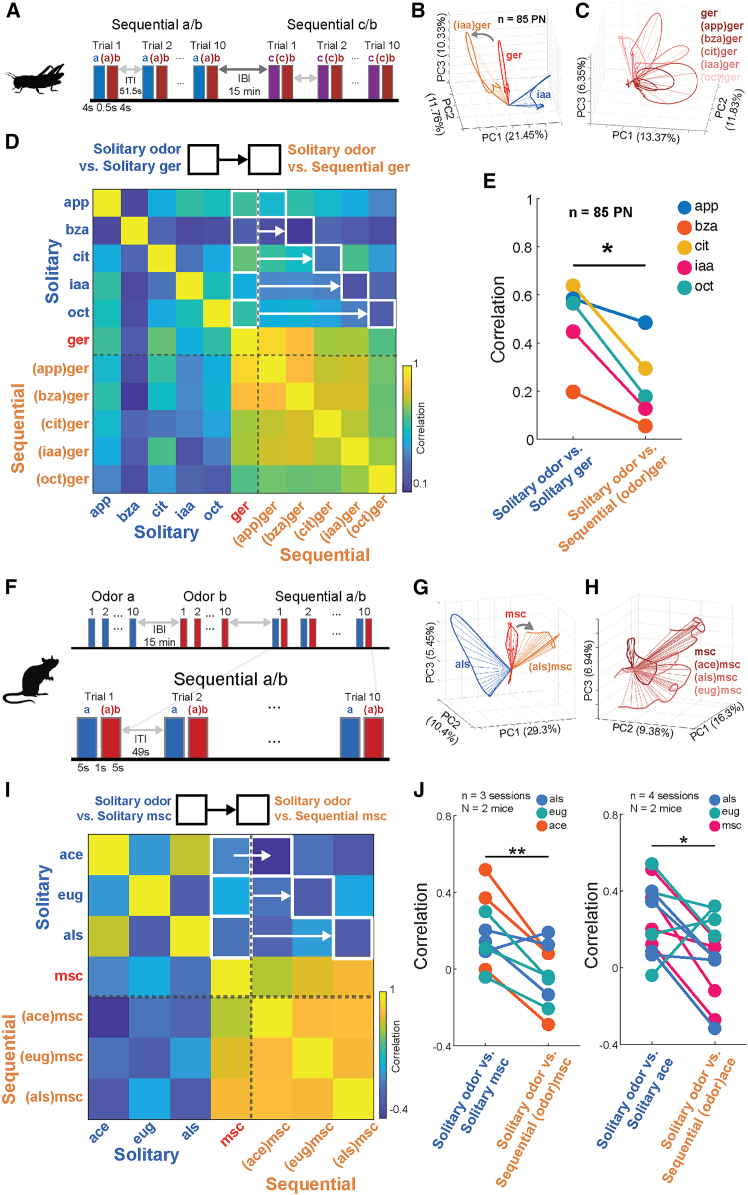
Contrast between sequential odors is enhanced (A) Experimental design for blocks of sequential odor presentations to locusts. Odors presented include geraniol (ger), isoamyl acetate (iaa), benzaldehyde (bza), citral (cit), octanol (oct), and apple (app). 4 s pulses of Odor A were presented with 4 s pulses of Odor B presented 0.5 s afterward during the OFF period of Odor A. (B) PCA trajectory ON responses to solitary iaa (blue), solitary ger (red), and ger sequentially after iaa ((iaa)ger, orange). (C) PCA trajectory of ger ON responses. Traces represent average PN response during the ON period to ger alone (ger), or ger with different odors presented immediately prior (sequential: (app)ger, (bza)ger, (cit)ger, (iaa)ger, and (oct)ger). (D) Correlation of average PN ON responses to solitary odors (app, bza, cit, iaa, oct, and ger) and to ger presented in sequence after other odors ((app)ger, (bza)ger, (cit)ger, (iaa)ger, and (oct)ger). White boxes and arrows indicate which correlation values are used for the quantitative comparison in (E). (E) Correlation values of PN population responses to solitary ger vs. PN population responses to other solitary odors (left boxes in D) compared to correlation values of PN responses to solitary odors vs. PN responses to ger presented sequentially after the other odors (right boxes in D, ∗*p* = 0.011, paired *t* test, *n* = 5 odor pairs). (F) Schematic of odor presentations to mice showing solitary presentations of single odors and sequential presentations of two odors with a 1 s interval between paired odors in a trial. Odors presented include acetophenone (ace), methyl salicylate (msc), eugenol (eug), and allyl sulfide (aLs). 5 s pulses of odor A were presented with 5 s pulses of odor B presented 1 s afterward during the OFF period of Odor A. (G) Representative PCA trajectories showing responses to solitary and sequential presentations of msc. solitary als (blue), solitary msc (red), and msc sequentially presented after als ((aLs)msc, orange). (H) Representative PCA trajectories showing the response to a solitary presentation of msc (dark red) and responses to msc sequentially presented after ace ((ace)msc), eug ((eug)msc), and als ((aLs)msc, lighter reds). (middle) solitary als (blue), solitary msc (red), and msc sequentially presented after als ((aLs)msc, orange). (right) solitary ace (blue), solitary msc (red), and msc sequentially presented after ace ((ace)msc, orange). (I) Correlation of glomerular ON responses across solitary presentations of eug, ace, msc, and als and sequential presentations of msc following ace ((ace)msc), eug ((eug)msc), and als ((aLs)msc). White boxes and arrows indicate correlation values used for the quantitative comparison in (J, left). (J) (left) Correlation values between solitary presentations of msc and other odors (left boxes in I) compared to correlation values between sequential msc and odors across imaging sessions (right boxes in D, ∗∗*p* = 0.004, paired *t* test, *n* = 9 odor pairs) (right). Correlation values between solitary presentations of ace and the three other odors compared to correlation values between sequential ace and odors across imaging sessions (∗*p* = 0.018, paired *t* test, *n* = 12 odor pairs).

Given that odors can be recognized within hundreds of milliseconds of stimulus onset,^28^ how can OFF responses, which begin after a stimulus has been terminated, contribute to odor processing? One possibility is that neural activity after stimulus offset could interfere with subsequent odor responses. To test this hypothesis, we presented both mice and locusts with pairs of odors in rapid succession, such that the OFF response of the first odor would overlap with the ON response of the second odor (Figures 4A and 4F). In locusts, we found that AL ensemble activity varied systematically as a function of prior odor exposure. For this, we compared PN neural responses evoked by a single odorant (geraniol, ger) encountered by itself (“solitary”) or as the second odor in a two-odor sequence (“sequential”). As expected, response trajectories evoked during the solitary (Figure 4B, red) versus the sequential geraniol presentations (Figure 4B, orange) differed significantly. Specifically, we found that geraniol responses during the sequential encounters became more distinct (greater angular separation) with respect to the first odor in that sequence (Figure 4B, blue). Also, presentations of geraniol after multiple other odors produced response trajectories that varied depending on the prior odor in the sequence (Figure 4C). This was observed for all odor sequences tested. Consistent with PCA trajectory analysis, we found that the correlation between high-dimensional odor response vectors was lower when the odors were sequentially presented, compared to when they were presented in isolation (Figure 4D). Across odors, coefficients were systematically higher between odor responses when presented in isolation (“solitary” condition) compared to when odors were presented sequentially (paired *t* test, *p* = 0.011, Figure 4E). Together, these data support the hypothesis that inverted OFF responses among locust PNs allow AL circuits to enhance response contrast between sequentially encountered odorants. Similar to locust experiments, we next presented mice with either solitary odors, or odors in sequence (Figure 4F). As observed for locust PNs, mouse glomerular odor response trajectories for sequentially presented odors reliably deflected away from trajectories of previously presented odors, indicating that responses to odors became more distinct during the sequential encounters (Figure 4G). For example, supporting the stimulus history dependence of mouse glomerular odor responses, presentations of the odorant methyl salicylate (msc) after multiple other odors produced response trajectories that varied depending on the prior odor in the sequence (Figure 4H). To quantify history-dependent contrast enhancement, we measured correlations between glomerular odor responses when odors were presented to mice sequentially or in isolation. Similar to what we observed in locust, the correlation between high-dimensional odor response vectors was lower when the odors were sequentially presented, compared to when they were presented in isolation (Figure 4I). In fact, correlation coefficients were systematically higher when odors were presented in isolation (“solitary”) compared to when odors were presented sequentially (msc as reference odor: paired *t* test, *p* = 0.004; ace as reference odor: one-tailed *t* test, *p* = 0.018), a finding which generalized across odor sequences (Figure 4J). Also, using qualitative trajectory analyses, together with the high-dimensional correlation analyses, we found that when odors were presented sequentially, responses in the mouse OB became less similar. Together, our locust and mouse results suggest that contrast enhancement by inverted odor OFF responses represent a computation of early olfactory circuitry that is conserved across species.

### Odor OFF responses persist long after odor offset

**Figure 5:**
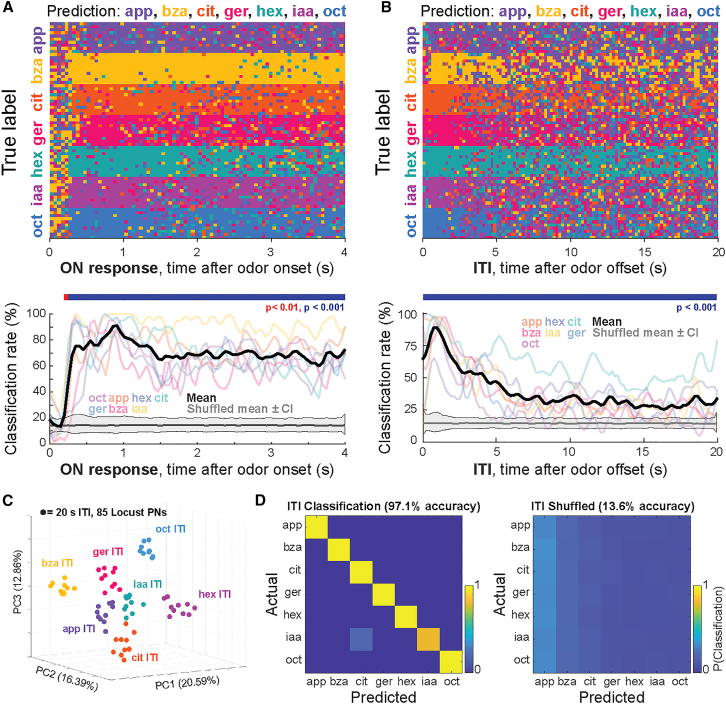
Odor-specific OFF responses persist in the locust AL (A) (top) Time-bin-by-time-bin k-NN classifier (k = 10; correlation distance; locusts PN activities) predictions for the odor presentation ON period time bins are shown. Odor predictions at each time point are color coded. (bottom) Quantification of classifier accuracy during the odor presentation ON period for each odor (colored transparent lines) and averaged across odors (black line). Shuffled data mean and confidence intervals are shown in gray. Colored bars indicate time points at which decoding exceeded chance based on a one-sided permutation test after Benjamini-Hochberg false discovery rate (BH FDR) correction (q = 0.05). (B) (top) Time-bin-by-time-bin k-NN classifier (k = 10; correlation distance; locusts PN activities) predictions for the post stimulus ITI time bins color coded by odor. (bottom) Quantification of classifier accuracy during the ITI time window for each odor (colored transparent lines) and averaged across odors (black line). Shuffled data mean and confidence intervals are shown in gray. Colored bars indicate time points at which decoding exceeded chance based on a one-sided permutation test after BH FDR correction (q = 0.05). (C) Dimensionality-reduced ensemble PN activity during 20 s after odor offset during intertrial intervals (ITI) between presentations of the same odor color coded by odor. (D) (left) Results from a K-NN classification analysis of PN activity using angular separation of full-dimensional data as the distance metric. (right) Average classification over 1000 permutations of shuffled data using angular separation of full-dimensional data as the distance metric.

OFF responses in locust AL and mouse OB represent the persistence of odor-specific information after the termination of the stimulus, suggesting a neural basis for a form of short-term memory. This raised the question of how long, and in what form, odor information persists in olfactory neuronal networks. To examine this, we tested whether and for how long odor identity could be inferred based on stimulus-evoked and post-stimulus ensemble activity using a linear classifier. As expected, classifier performance rapidly increased at odor onset and remained elevated for the duration of the odor presentation in both locusts and mice (Figure 5A). After odor offset, we found that among locust PNs, odor information decayed gradually across the intertrial interval (ITI). Classifier accuracy decreased to ∼40% across odors throughout the 20-s post stimulus ITI, but notably, for most odors, accuracy did not fall to chance levels during the entire ITI period (Figure 5B). Complementing the classification analysis over time, we performed a time-averaged classification analysis spanning the entire ITI period. Using PCA analysis to visualize post-stimulus neural activity during the entire ITI, we found that time-averaged classifications clustered robustly by odor identity (Figure 5C). Further, a k-nearest neighbor classifier incorporating angular distances of full-dimensional ITI data robustly discriminated odors above chance levels (Figure 5D). Together, these analyses supported the interpretation of the PCA analysis and showed that ITI activity was odor specific.

**Figure 6:**
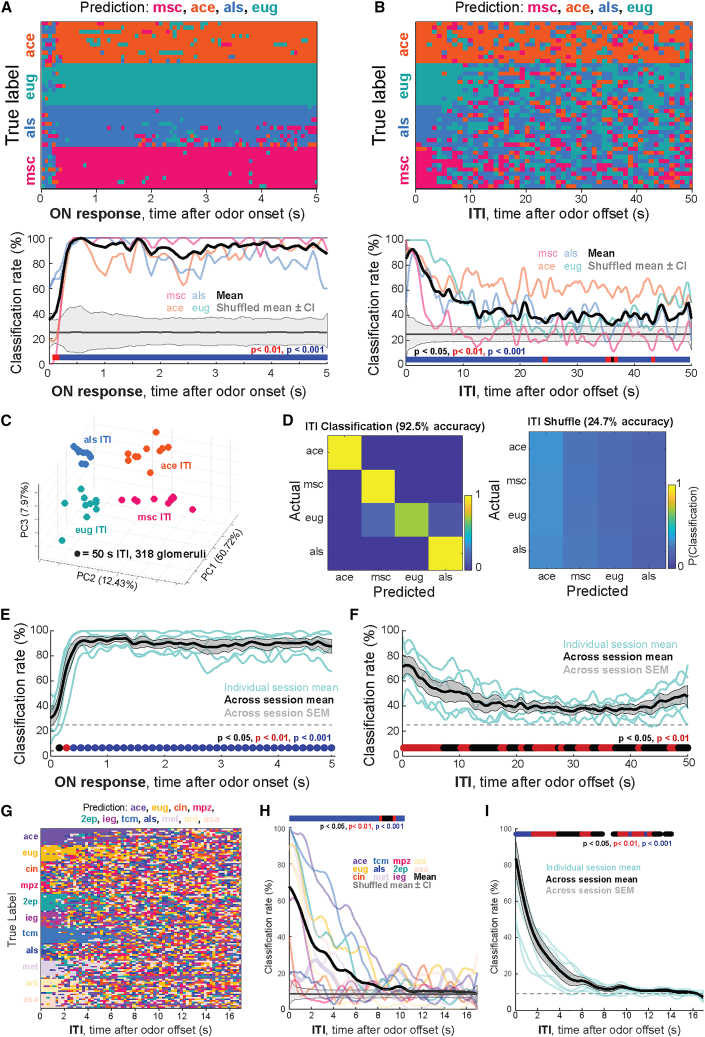
Odor-specific OFF responses persist in the mouse OB (A) (top) Time-bin-by-time-bin k-NN classifier (*k* = 10; correlation distance; mouse glomerular activities) predictions for the odor presentation ON period time bins are shown. Odor predictions at each time point are color coded. (bottom) Quantification of classifier accuracy during the ON period for each odor (colored transparent lines) and averaged across odors (black line). Shuffled data mean and 95% confidence intervals of the null distribution are shown in gray. Colored bars indicate time points at which decoding accuracy exceeded chance following Benjamini-Hochberg false discovery rate (BH FDR) correction (q = 0.05). (B) (top) Time-bin-by-time-bin k-NN classifier (*k* = 10; correlation distance; mouse glomerular activities) predictions of the post stimulus ITI time color coded by odor. (bottom) Quantification of classifier accuracy during the ITI time window for each odor (colored transparent lines) and averaged across odors (black line). Shuffled data mean and 95% confidence intervals of the null distribution are shown in gray. Colored bars indicate time points at which decoding accuracy exceeded chance following BH FDR correction (q = 0.05). (C) Dimensionality-reduced mouse OB glomerular activity during the 55 s ITIs between presentations of the same odor within a block of 10 trials color coded by odor. (D) (left) Results from a K-NN classification analysis of the glomerular activity (k = 20) during the 55 s ITI period, based on angular distance of full-dimensional data. (right) Average classification over 1000 permutations of shuffled based on angular distance of full-dimensional data. (E) Quantification of average classifier accuracy across odors for each session during the ON period (blue transparent lines, *N* = 6 sessions from 3 mice) and averaged across sessions (black line). Gray shows ±SEM. Colored circles indicate binned time points at which decoding exceeded chance based on a one-sided *t* test with BH FDR correction. (F) Quantification of average classifier accuracy across odors for each session during the ITI (blue transparent lines, *N* = 6 sessions from 3 mice) and averaged across sessions (black line). Gray shows ±SEM. Colored circles indicate binned time points at which decoding exceeded chance based on a one-sided *t* test with BH FDR correction. (G) Mouse data with eleven odors presented in a random order. Odors included acetophenone (ace), eugenol (eug), cineole (cin), methypyrazine (mpz), 2-ethylpyrizine (2ep), isoeugenol (ieg), transcinnamaldehyde (tcm), allylsufide (aLs), methylsalycylate (met), anisole (ani), and isoamyl acetate (asa). Time-bin-by-time-bin k-NN classifier (*k* = 10; correlation distance; mouse glomerular activity) predictions of the post stimulus ITI time bins. Odor predictions at each time point are color coded. (H) Quantification of classifier accuracy during the ITI time window for each odor (colored transparent lines) and averaged across odors (black line) for a single session. Shuffled data mean and 95% confidence intervals of the null distribution are shown in gray. Colored bars indicate time points at which decoding accuracy exceeded chance following BH FDR correction (q = 0.05). (I) Quantification of average ITI classifier accuracy across odors for different sessions (blue transparent lines, *N* = 8 sessions from 6 mice) and averaged across sessions (black line). Gray shows ±SEM. Colored circles indicate binned time points at which decoding exceeded chance based on a one-sided *t* test with BH FDR correction.

We next tested the extent to which odor-specific information persisted in the mouse OB and whether its temporal decay differed from that observed in locusts. Strikingly, we found that odor information persisted for even longer in the mouse OB. As in locust, classifier accuracy rapidly increased and remained high for the duration of odor presentations (Figure 6A). However, after odor offset, we found that classifier accuracy remained high for the duration of the 50-s ITI (Figure 6B). We observed odor-specific clustering of 55-s ITI activity from the mouse OB (Figure 6C), which contributed to highly accurate classification of odors from ITI glomerular responses, similar to what we observed in locusts (Figure 6D). Additionally, overall classifier accuracy was consistently high across mice and imaging sessions during the odor presentation period (Figure 6E) and remained above chance for the duration of the ITI after odor offset (Figure 6F, *N* = 6 sessions from 3 mice). Finally, to determine whether odor information persisted in the ITI after single presentations of an odor, as opposed to repeated presentations of the same odor, we presented eleven odors in a random order and measured classifier performance throughout the ITI. In these experiments, odors were presented for 1 s, with 19 s ITIs and no block structure. Classifier accuracy across the ITI was variable across odors, but, on average, declined to chance after 10 s (Figure 6H). Furthermore, overall classifier accuracy remained above chance for 10–15 s after odor offset across mice and sessions (Figure 6I, *N* = 8 imaging sessions from 6 mice). This contrasted with the repeated odor presentation condition, where accuracy remained above chance for the duration of the 55 s ITI. The difference in the persistence of odor information across experiments may be explained by accumulating hysteresis when odors are presented repetitively. This would suggest ongoing, odor-specific local circuit activity after odor offset, though further experiments are necessary to determine the nature and circuit mechanism of the hysteresis. Nevertheless, these findings collectively indicate that both AL PNs and OB glomeruli maintain information about an odor stimulus well after its termination.

## Discussion

The primary goal of this study was to identify common computational principles that govern the initial stages of olfactory processing across distinct species. Toward this we employed a comparative approach, utilizing calcium imaging in the mouse OB and electrophysiological recordings in the locust AL, leveraging the unique strengths of each model and recording modality. In the mouse OB, genetically targeted calcium indicators and two-photon imaging allowed us to isolate and image postsynaptic dendritic responses across the glomerular layer. Due to the rigidly stereotyped anatomy of mouse OB circuits, these dendritic responses were easily grouped into glomerular units comprised of highly correlated sister mitral cells.^29^^,^^30^ Thus, calcium imaging from dendrites in the glomerular layer of the mouse OB provided a way to pool signals from functionally related mitral cell cohorts while sampling a broad population of odor responsive neurons postsynaptic to OSNs.^31^ At the same time, single cell electrophysiology in the locust provided a complimentary window into odor response dynamics by providing high temporal resolution information about action potential firing in individual cells. While just a few units were simultaneously recoded in locust AL, population odor responses were collected across multiple experiments. As such, multi-unit recordings in locusts allowed us to record from AL PNs without genetic targeting and quantify AL PN output in the form of action potential firing downstream of complex sensory neuron input, local circuit modulation, or intrinsic changes that might occur at the cell body.

Despite differences in the population breadth and kinetics of electrophysiological vs. imaging modalities, as well as the structural differences in locust and mouse olfactory systems, our comparative approach revealed surprisingly conserved odor response dynamics across species. First, we found that responses to odor onset (ON responses) and odor offset (OFF responses) were distinct and anticorrelated. We observed modest correlations across odors during the ON period, within the range commonly reported by other studies.^21^^,^^32^^,^^33^^,^^34^ We also noted that OFF responses interfere with subsequent odor presentations and enhance contrast between sequentially presented odors in both mouse and locust. Finally, we found that inverted OFF responses persist and maintain short-term odor memories over several seconds. Ultimately, our comparative approach revealed functional consequences of long-lasting, temporally complex odor-evoked activity that seems to generalize across species, even if the quantitative details of these effects are different, and likely influenced by recording modality, model, and experimental design. Taken together, our findings support the notion that odor response dynamics are conserved across systems to serve core functions of early olfactory circuits in both the locust AL and mouse OB. While general sensory aftereffects and the persistence of neural activity following stimulus termination are known phenomena in various sensory systems, including olfaction,^23^ our work reveals a unique form of post-stimulus activity where responses evoked by odor onset are anticorrelated with those evoked by odor offset.

Persistently altered neural dynamics and distortions in perception following a stimulus, known as “aftereffects”, are a ubiquitous feature of sensory systems across species. Aftereffects correspond to changes in neuronal activity with stimulus offset, either through neuronal adaptation that occurs during stimulus presentation, or through distinct stimulus offset-evoked responses.^35^^,^^36^^,^^37^^,^^38^^,^^39^^,^^40^^,^^41^ Importantly, perceptual distortions of aftereffects reveal computations performed by sensory circuits that are critical for stimulus detection and discrimination. In olfaction, aftereffects have been described in the form of distinct neural responses to the offset of odors in worms, insects, fish, and mammals.^23^^,^^27^^,^^42^^,^^43^^,^^44^ Prior studies in insect and mammalian olfactory systems have described odor-evoked OFF responses and post-odor inhibitory dynamics in both insect and mammalian olfactory systems.^45^^,^^46^ Additionally, in the mammalian olfactory system, previous studies have shown that odor-specific information briefly persists in the OB after stimulus offset.^23^ Building on this prior work, our findings reveal a structured inversion of OFF responses at the population level, whereby post-odor activity systematically mirrors ON responses, forming an anti-correlated representational geometry. Additionally, we found that patterns of glomerular activity persisted for several seconds after individual odor presentations, and for the entire duration of the ITI when odor presentations were repeated, allowing odor identification for up to a minute after exposure. Our results build on previous studies to suggest that odor memories, in the form of after images, are longer lasting than previously appreciated. Notably, the anticorrelated structure of OFF responses in mice and locusts arises from the suppression of ON responding neurons and glomeruli during the OFF period, and the suppression of OFF responding neurons and glomeruli during the ON period. At the same time, distinct sets of neurons were activated during ON and OFF periods. Whether this is a generic strategy for retaining stimulus specific information in neuronal networks over time, how much stimulus specific information is encoded in the suppression of ON responders vs. the activation of OFF responders, and whether complementarity in activated sets of neurons also forms the basis for other adaptive computations remains unknown. Importantly, however, the current work reveals a specific form of persistent population neural activity, anticorrelated to odor-evoked responses, which allows information about recently encountered odors to influence subsequent odor responses.

An intriguing and critical question is what cellular and circuit mechanisms underpin these conserved, anticorrelated OFF responses, particularly given the significant evolutionary divergence and architectural differences between insect and mammalian olfactory systems. In both insect and mouse sensory neurons, inhibitory responses to odors have been observed.^47^^,^^48^^,^^49^ However, sensory neuron inhibition is unlikely to be the sole cause of the observed OFF response anticorrelations given the inability to explain downstream suppression of otherwise active glomeruli, suppression of glomeruli after odor offset, or rebound excitation of glomeruli after suppression during odor presentations, and, in some cases, it relies on presenting odors as complex mixtures.^50^ In locusts, the AL circuit involves interactions between cholinergic PNs and GABAergic LNs. Previous work, corroborated by our findings, has shown that distinct and anticorrelated OFF responses in locust PNs are generated through a combination of cell-intrinsic properties, such as adaptive changes in neuronal spike thresholds, and local GABAergic circuit interactions with inhibitory interneurons.^27^ The inverted OFF responses, in large part, rely on adaptive changes in spike-thresholds of individual PNs, such that PNs responding highly to an odor have elevated spike thresholds and are less-likely to respond at odor offset. At the same time, interconnectivity of PNs with local inhibitory interneurons also contributes to enhancing inverted OFF responses.^51^ Adaptation at the level of sensory neurons and activity-dependent synaptic depression between sensory neurons and PNs may contribute to shaping these dynamic responses. However, the emergence of strong, orthogonal OFF responses appears to be a key transformation occurring within the AL circuit beyond the ORN input, where pure OFF responses are seldom observed. Our current results suggest that OFF responses in mice are analogous to OFF responses in insect models both structurally and functionally. Considering circuit mechanisms underlying anticorrelated OFF responses, mitral and tufted cells (MTCs) in mice are analogous to locust PNs and exhibit similar features of intrinsic adaptation.^39^ MTCs are also highly interconnected with both local inhibitory granule cells and periglomerular interneurons.^52^ Our results suggest that OFF responses in the mouse OB may be caused by the release of odor-evoked inhibition mediated by these local inhibitory circuits. While sensory neuron activity largely ceases after odor offset, the complex dynamics observed in MTCs, including the transition from ON excitation to OFF activation or inhibition, cannot be solely explained by simple sensory adaptation. Thus, similar cellular and circuit mechanisms may underlie the inversion of odor OFF responses in mouse and locust models, though future studies will be required to determine the extent to which this is true.

It is crucial, however, to highlight a major difference in the overall architecture of these two systems: unlike the largely feedforward structure of the insect AL, the mammalian OB receives extensive centrifugal feedback projections from the olfactory cortex and is modulated by top-down cholinergic and noradrenergic inputs.^10^^,^^11^^,^^12^^,^^13^^,^^53^^,^^54^^,^^55^^,^^56^ Multiple lines of evidence suggest that this central feedback plays a significant role in shaping OB activity and potentially maintaining persistent neural representations.^23^ It has been proposed that persistent odor OFF responses in mice might be centrally maintained or influenced by this cortical feedback. Indeed, these differences in large-scale circuit mechanisms, particularly the presence of extensive cortical feedback in mice, may contribute to quantitative differences observed between the species, such as the potentially greater degree of anticorrelation, or longer persistence of the OFF response in mouse OB compared to locust AL. The enhanced feedback in the mouse system could, for example, actively drive the inverted OFF responses, leading to a stronger anticorrelation, though future work is required to test this hypothesis. Nevertheless, the surprising qualitative conservation of the anticorrelated ON-OFF population dynamics across species strongly suggests that analogous mechanisms drive key odor processing dynamics in both mouse and locust early olfactory centers. Furthermore, this conservation implies that generating an inverted odor afterimage and utilizing it for subsequent processing are fundamental computations in olfactory systems, achieved potentially through convergent mechanisms that have coopted similar local circuit motifs within vastly different global network structures.

Building on conserved computational roles of the anticorrelated ON-OFF dynamics, a crucial remaining question is regarding the direct impact on olfactory perception and odor-guided behavior. In locusts, compelling evidence suggests that odor OFF responses are behaviorally meaningful. Previous work links ON responses to “sensing” behaviors and OFF responses to “unsensing” behaviors, such as the closing of palps.^27^ Specifically, a flexible decoding model successfully predicted palp-opening responses to sequential odors and accurately predicted instances where ON and OFF responses canceled each other out.^57^ This model provides strong support that the distinct and orthogonal nature of the ON and OFF representations is actively utilized by downstream circuits to influence olfactory behavior in locusts. Additionally, a recent study used behavioral experiments in locust to demonstrate that population odor response trajectories for different odors cluster according to innate and learned valences of odors, indicating that population response structure is related to high-level features of odor perception.^58^ While these data suggest that OFF responses are behaviorally meaningful in locusts, no evidence currently exists to suggest how odor OFF responses impact mouse olfactory perception. While it has been suggested that olfactory afterimages in mice may facilitate the identification of novel odors in complex environments,^23^ future work is required to determine precisely how odor perception in mice is shaped by odor OFF responses, and whether these effects impact odor-guided behavior. Broadly, the demonstration of a conserved odor afterimage in both locusts and mice underscores its potential as a fundamental olfactory computation. However, translating these neural dynamics and computational roles into a full understanding of their perceptual consequences and direct behavioral impact remains a critical direction for future investigation, particularly in the mammalian system where the link between the neural afterimage and olfactory guided behavior is not yet established.

### Limitations of the study

One limitation of the current study is the use of different recording modalities including electrophysiology in locusts, which captures spike-level activity, and glomerular calcium imaging in mice which indir ectly reports dendritic activity from a population of neurons. Thus, we are comparing neural dynamics across different populations and at different temporal resolutions. Nevertheless, anti-correlated ON-OFF patterns emerge across the different modalities and species, which suggest that the phenomenon is fundamental rather than an artifact of recording technique. A second limitation is that, while we demonstrate that anti-correlated ON-OFF dynamics enhance neural contrast at the circuit level, we have not established their necessity for behavioral odor discrimination. Future work should include perceptual experiments testing whether odor discrimination performance differs for sequential versus isolated presentations, optogenetic manipulation of OFF responses during discrimination tasks, and behavioral tests to determine whether perceptual aftereffects match neural timescales. A third limitation is that the current study does not define specific cellular and circuit mechanisms underlying anti-correlated ON-OFF dynamics. Multiple mechanisms including local inhibition, adaptation, and in mammals centrifugal feedback could contribute to these patterns. Targeted manipulations using cell-type-specific optogenetics, pharmacological receptor blockade, and disruption of feedback projections are needed to distinguish among these possibilities. Finally, our findings are based on sets of monomolecular odors at fixed concentrations. Whether ON-OFF anti-correlation persists for odor mixtures, scales across concentration ranges, or extends to naturalistic stimuli remains to be tested. Future experiments examining complex mixtures, concentration-dependent effects, and ethologically relevant odors would reveal the full scope of this phenomenon and reveal whether its magnitude or duration depend on the nature of the odor stimuli. Despite these limitations, the convergent finding of anti-correlated ON-OFF dynamics across two evolutionarily distant species using fundamentally different experimental approaches provides strong evidence that this represents a conserved computational principle in early olfactory processing, with the limitations pointing toward exciting avenues for mechanistic and functional investigation in the future.

## Resource availability

### Lead contact

• Further information and requests for resources and reagents should be directed to the lead contact Baranidharan Raman, barani@wustl.edu.

### Materials availability

• This study did not generate new reagents.

### Data and code availability

• Locust electrophysiology and mouse calcium imaging data is publicly available at Figshare: https://doi.org/10.6084/m9.figshare.31435543. DOI is listed in the key resources table.
• Original code used to analyze data and generate figures is publicly available at Figshare: https://doi.org/10.6084/m9.figshare.31435543. DOI is listed in the key resources table.
• Any additional information required to reanalyze the data reported in this paper is available from the lead contact upon request.

## Acknowledgments

We thank Dr. Fabrizio Gabbiani for feedback on the manuscript, Pearl Olsen for insect care, and Evelyne Tantry and Elaine Le for mouse care. This research was supported by 10.13039/100000001NSF (1724218 and 2021795) and 10.13039/100000006ONR (N00014-19-1-2049, N00014-21-1-2343) grants to B.R., 10.13039/100000002NIH (R00DC019505) to E.H.M., the McNair Medical Institute, 10.13039/100000002NIH awards (10.13039/100000065NINDS R01NS078294), (10.13039/100000062NIDDK R01DK109934), and 10.13039/100000005DOD (PR180451-PRMP) awards to B.R.A., and 10.13039/100000002NIH awards (10.13039/100000065NINDS UF1NS111692) to B.R.A. and J.R.

## Author contributions

Conceptualization: B.R.A. and B.R.; methodology: D.L., E.H.M., B.R.A., B.R., and J.R.; formal analysis: D.L., F.D., and B.R.; investigation: D.L., E.H.M., and R.K.; data curation: D.L., B.R., E.H.M., C.L.S., and J.R.; writing – original draft: D.L. and E.H.M., writing – reviewing and editing: B.R.A. and B.R.; visualization: D.L., E.H.M., and F.D.; supervision: B.R.A., B.R., and J.R.; project administration: B.R.; funding acquisition: E.H.M., J.R., B.R., and B.R.A.

## Declaration of interests

The authors declare no competing interests.

## STAR★Methods

### Key resources table

**Table:** 

REAGENT or RESOURCE	SOURCE	IDENTIFIER
**Chemicals, peptides, and recombinant proteins**		

Mineral Oil	Sigma	Cat# M8419
Acetophenone	Sigma	Cat# A10701
Allyl Sulfide	Sigma	Cat# W204218
Eugenol	Sigma	Cat# E51791
Methyl Salicylate	Sigma	Cat# W274518
Isoamyl Acetate	Sigma	Cat# W205508
Cineole	Sigma	Cat# C80601
Methypyrazine	Sigma	Cat# M37702
2-Ethypyrazine	Sigma	Cat# E85007
Isoeugenol	Sigma	Cat# W246608
*Trans*-cinnamaldehyde	Sigma	Cat# C80687
Anisole	Sigma	Cat# A41501
Ammonia	Sigma	Cat# 221228
Apple	Sigma	Cat# W248205
Benzaldehyde	Sigma	Cat# B1334
Citral	Sigma	Cat# W230217
Geraniol	Sigma	Cat# 163333
1-Hexanol	Sigma	Cat# W278116
Hexanoic Acid	Sigma	Cat# W247708
1-Nonanal	Sigma	Cat# N42008
(Z)-3-Nonen-1-ol	Sigma	Cat# 322387
Nonanoic Acid	Sigma	Cat# N5505
Octanoic Acid	Sigma	Cat# W261401
1-Octanol	Sigma	Cat# 01380
Phenylethylamine	Sigma	Cat# 13080
4-Vinyl anisole	Sigma	Cat# W369600

**Deposited data**		

Locust electrophysiology data	Figshare	https://doi.org/10.6084/m9.figshare.31435543
Mouse two-photon imaging	Figshare	https://doi.org/10.6084/m9.figshare.31435543

**Experimental models: Organisms/strains**		

*Mus musculus* (mouse), Thy1-GCaMP6f (line GP5.11)	Jackson Laboratory	JAX #025393
*Schistocerca americana* (locust)	In-house colony	–

**Software and algorithms**		

ScanImage	Vidrio	RRID: SCR_014307
MATLAB	Mathworks	RRID: SCR_001622
LabView	National Instruments	RRID: SCR_014325
Analysis code	Figshare	https://doi.org/10.6084/m9.figshare.31435543

### Experimental model and study participant details

#### Mice

All experimental procedures were approved by the Baylor College of Medicine licensed Institutional Animal Care and Use Committee under protocol number AN5596. Thy1-GCamp6f (GP5.11, Jax laboratories #025393) mice, age two months to four months, of both sexes, were used for two-photon imaging experiments. Line GP5.11 has been well-described by multiple groups to express GCaMP6f robustly in mitral and tufted cell dendrites in the glomerular layer, with minimal expression in interneurons.^31^^,^^59^^,^^60^^,^^61^^,^^62^^,^^63^ Experiments were conducted in awake, head fixed mice receiving passive odor presentations. Mice were housed in a standard 12-h light/dark cycle and had *ad libitum* access to food and water.

#### Locusts

Post-fifth instar adult locusts (*Schistocerca americana*) were reared in a crowded colony with a 12-h light-dark cycle. Both male and female were used for electrophysiological experiments.

### Method details

#### Cranial window surgery

Chronic cranial windows were created in mice by removing a 4 mm diameter section of skull over the OB and inserting a glass coverslip. Before surgery mice were treated with 5 mg/kg Meloxicam. Anesthesia was induced and maintained with isoflurane during the surgical procedure. After induction of anesthesia, the scalp was injected subcutaneously with 0.05 mL bupivacaine, then cleaned and removed over the OB and dorsal skull. A 0.16″ thick stainless-steel shim (McMaster-Carr, A370-974) was centered over the OB and attached to the exposed skull with dental cement (C&B Metabond). A 4 mm diameter piece of skull was removed by carefully drilling though the skull. A 4 mm glass coverslip (Warner instruments) was placed over the exposed brain and sealed in place with tissue adhesive (3M Vetbond). The sealed window was then stabilized with high-viscosity cyanoacrylate superglue (Loctite). After the glue fully cured, the coverslip was protected with a cap of Kwik-Cast silicone elastomer (World Precision Instruments) which was removed immediately before imaging.

#### Odor delivery to mice

All imaging was performed on head-fixed, awake mice on a running wheel as they were passively exposed to odors. Mice were head fixed using a custom headplate^47^ designed to attach to the stainless-steel shim implanted on the skull during the cranial window surgery. Mice were habituated to the head fixation and imaging setup for at least 30 min prior to testing each day. For passive odor delivery, a custom-built, multi-channel olfactometer^48^ was placed 6 cm in front of the mouse. The olfactometer provided a constant stream of room air into which experimental odors were injected. Odors were mixed into the central airstream by an eductor positioned at the output of the olfactometer before delivery to the mouse. Odors for mouse imaging experiments were obtained from Sigma and included methyl salicylate (msc), allyl sulfide (aLs), acetophenone (ace), and eugenol (eug), diluted to 10% by volume in mineral oil. Odors were further diluted to ∼1% of their initial concentration by injection into the central airstream (∼8 L/min flow rate). Odor delivery was controlled and synchronized with imaging via custom LabVIEW software. The timing of odor onset and offset was verified with PID recordings (200B miniPID, Aurora Scientific) made adjacent to the mouse nostril. Odors were scavenged after delivery by a vacuum positioned 10 cm behind the mouse in line with the air stream of the olfactometer.

#### Odor presentation paradigms

For solitary odor presentation experiments, odors were delivered passively for 5 s via injection into the central air stream. Each odor was repeated 10 times in a block with a 55 s intertrial interval between presentations within a block and 15 min between blocks of different odors (Figure 1B). Mice were presented with each odor bock once in an imaging session so that they experienced four total odor blocks and 40 total odor presentations. For sequential presentation experiments, following solitary odor presentations, odors were presented in pairs such that the first odor was presented for 5 s followed by a 1 s gap and then a 5 s presentation of the second odor (Figure 4F). Odor pairs were presented in blocks of 10 with 49 s between trials within a block and 15 min between blocks of different odor pairs. Each mouse was presented 3 blocks of pairs, for a total of 30 pair trials per session in a 2 h and 40-minute-long session. In separate mice, for random order odor presentations (Figure 6H), odors were presented for 1 s with a 19 s intertrial interval. 11 different odors and mineral oil were presented in a random order over 480 trials with no block structure so that each odor was presented 20–60 times in a 2 h and 40-minute-long session. Only the first 10 presentations of each odor were used for classification analyses to allow comparisons with block-structured experiments. For experiments using 11 odors and mineral oil, odors included acetophenone (ace), eugenol (eug), cineole (cin), methylpyrazine (mpz), 2-ethylpyrizine (2ep), isoeugenol (ieg), trans cinnamaldehyde (tcm), allyl sufide (aLs), methyl salicylate (met), anisole (ani), and isoamyl acetate (asa), all diluted to 1:10 by volume and further diluted ∼1:100 during delivery.

#### Two-photon imaging

Two-photon imaging was performed on a ThorLabs/Janelia 2P-RAM mesoscope.^28^ The laser wavelength was set to 920 nm to image GCaMP6f signals. Imaging parameters were controlled with ScanImage software. To maximize frame rates in each imaging session, fields of view were defined for acquisition that included a single plane visualizing only the dorsal surface of bilateral OBs (1800 μm long x ∼600μm –wide fields of view that were tiled to cover a total field of view that was 1800–2500 μm wide). Images were acquired continuously throughout experiments (during odor presentations, intertrial, and inter-block intervals), with 5um/pixel resolution at the fastest possible frame rate allowed by the imaging parameters (15–18 Hz). After imaging, videos were motion and raster-corrected and glomerular ROIs were manually defined with custom software. Following definition of glomerular ROIs, fluorescence traces were extracted from ROIs, imported to MATLAB, converted to dF/F, and median filtered to remove any persistent motion artifacts.

#### Locust electrophysiology

Locusts were immobilized with both antennae intact. Then the primary olfactory region of their brain, the antennal lobe (AL), was exposed, desheathed, and perfused with room temperature saline. Extracellular multiunit recordings of projection neurons (PNs) were performed with a 16-channel, 4x4 silicon probe (NeuroNexus) that was superficially inserted in the AL. Prior to each experiment, all probes were electroplated with gold to achieve impedances in the range of 200–300 kΩ. The recordings were acquired with a custom 16-channel amplifier (Biology Electronics Ship; Caltech, Pasadena, CA). The signals were amplified with a 10k gain, bandpass filtered (0.3–6 kHz), and sampled at 15 kHz using a LabView data acquisition system. A visual demonstration of this protocol is available online.^49^

#### Odor delivery to locusts

Odor stimuli were delivered using a standard protocol previously described.^45^ The following odor panel was used for electrophysiological experiments: ammonia (amm), apple (app), benzaldehyde (bza), citral (cit), geraniol (ger), hexanol (hex), hexanoic acid (hxa), isoamyl acetate (iaa), 1-nonanal (nan), z-3-nonen-1-ol (nen), nonanoic acid (noa), octanoic acid (oca), octanol (oct), phenethylamine (phn), and 4-vinyl anisole (vny). All odors were diluted in mineral oil to 1% v/v concentration and sealed in 60 mL glass bottles with an air inlet and outlet. A pneumatic picopump (WPI Inc., PV-820) was used to deliver 0.1L/min of air to the odor bottle. The injected air displaced air containing diluted odorant in the bottle headspace, which was subsequently mixed with a desiccated 0.75L/min carrier air stream directed toward the locust antennae. A vacuum funnel placed behind the locust preparation continuously removed delivered odors. For solitary odor presentations, odors were presented in a pseudorandom order, in blocks of 10 trials (Figure 1A). For sequential presentation experiments, two odors were presented sequentially, separated by 500 ms. Pairs of odors were presented in blocks of 10 trials with the identity of the first order pseudorandomized and the second odor fixed (Figure 4A). Odors or odor pairs were presented with 60-s inter-trial intervals within blocks and 15-min intervals between blocks.

### Quantification and statistical analysis

#### Summary of locust and mice datasets

Two locust datasets were used to generate Figures 1, 2, 3, 4, and 5. The dataset used for generating Figures 4 and 5 were part of an earlier published dataset.^52^ Seven mouse datasets were used to generate Figures 1, 2, 3, 4, and 6. All datasets are summarized in Table 1.

#### PN spike sorting

To obtain single-unit PN responses, spike sorted was performed offline using four recording channels and conservative statistical principles.^50^ Spikes belong to single PNs were identified as described in earlier work.^51^ The following criteria were used to identify single units: cluster separation >5 x noise standard deviations, total number of spikes within 20 ms inter-spike interval <6.5% of total spikes, and spike waveform variance <6.5 x noise standard deviations. In total, 66 PNs from 15 locusts were identified for data shown in Figures 1, 2, and 3 and 85 PNs were identified from 9 locusts for data shown in Figures 4 and 5.

#### Time-bin-by-time-bin correlation analysis

Each pixel or matrix element in time-bin-by-time-bin correlation plots indicates the correlation value between neural activity vectors observed in the *i*^th^ and *j*^th^ time bins. All time-bin-by-time-bin correlation analyses were computed using high-dimensional response vectors. Correlations were calculated as:

(Equation 1) $$C_{ij}=\frac{cov\left(X_{i}^{ON},X_{j}^{OFF}\right)}{\sigma_{i}^{ON}\sigma_{j}^{OFF}}$$

Here, *X* is an n-dimensional activity vector, *i* and *j* represent time bins, $X_{i}^{ON}$ represents the population activity vector in the *i*^*th*^ time bin during the ON period, $X_{j}^{OFF}$ represents the population activity vector in the *j*^*th*^ time bin during the OFF period, $\sigma_{i}^{ON}{,\sigma }_{j}^{OFF}$ are standard deviations of spiking activities during ON and OFF periods during the i^th^ and j^th^ time bins respectively.

For locust data, PN spikes were binned in 50 ms non-overlapping time bins, and spike counts of different PNs were concatenated to obtain an n-dimensional population spike count vector (where *n* = 66 PNs for Figure 2B). For mouse data, dF/F values were taken to be an n-dimensional population glomerular activity vectors (where *n* = 198 glomeruli for Figure 2G).

#### Time averaged correlations analysis

All time averaged correlation analyses were computed using high-dimensional response vectors. Correlations were calculated as:

(Equation 2) $$C_{ij}=\frac{cov\left(X_{i}^{ON},X_{j}^{OFF}\right)}{\sigma_{i}^{ON}\sigma_{j}^{OFF}}$$

Here, *X*^*ON*^,*X*^*OFF*^ are trial- and time-averaged high-dimensional activity vectors for the ON and OFF periods, respectively, respectively.

For locust data, PN spikes during the 4 s odor stimulation were summed and averaged across ten trials to obtain an n-dimensional population PN average spike count vector (where: *n* = 66 PNs for Figure 3A; *n* = 85 PNs for Figure 4D). For mouse data, dF/F values were averaged across the 10 trials, and then averaged across the frames for the 5 s odor stimulation duration to obtain an n-dimensional population glomerular activity vector (where *n* = 198 glomeruli for Figure 3E; *n* = 219 glomeruli for Figure 4I).

#### Visualization of response trajectories through PCA

To visualize high dimensional AL PN spike counts and OB glomerular dF/F signals, we used a linear principal component analysis. For locust data, the spike counts for each PN in 50 ms time bin during the 4 s stimulation window were averaged across trials and concatenated across PNs to generate a *n*-dimensional vector (where *n* = 66 PNs for Figure 2C; trial-averaged neural trajectories). The high-dimensional PN spike count vector was projected onto the top three eigenvectors of the data covariance matrix.

For mouse data, dF/F values at each imaging frame (captured at 15–19 Hz) during the 5 s stimulation window were averaged across trials and concatenated across glomeruli to generate an n-dimensional vector (where *n* = 198 glomeruli for Figure 2H). The high-dimensional glomerular response vector was projected onto the top three eigenvectors of the data covariance matrix.

#### Visualization of ITI neural activity vectors through PCA

To visualize high dimensional AL PN spike counts and OB glomerular dF/F signals during intertrial intervals (ITI) and inter-block intervals (IBI) we used a linear principal component analysis. For locust data, the ITI was defined as the 21 s immediately following the odor offset. The spike counts for each PN were averaged across the 20 s ITI period. The resulting n-dimensional PN spike count vector (where *n* = 85 PNs for Figure 5C) was computed for each trial and visualized after PCA dimensionality reduction. The data points were colored based on the odor that preceded the ITI analysis window. For mouse data, the ITI was defined as the 50 s immediately following the odor offset. The dF/F values were averaged across the 50 s ITI period for each glomerulus and the resulting n-dimensional glomerular activity vector (where *n* = 318 glomeruli for Figure 6C) represented the glomerular activity following the odor offset for a given trial. Glomerular activity following odor termination in different trials were visualized after PCA dimensionality reduction, and the data points were colored based on the odor that preceded the ITI period.

#### Comparing angles between high-dimensional response vectors

Vector angles were calculated between trial-averaged PN activity for each 50 ms time bin in the 4 s ON period (Figures 2D and 2E). The resulting vector angles were subsequently binned according to the ON-ON, ON-OFF, or OFF-OFF period depending on the time window from which the PN vectors were picked. A similar analysis was carried out for mouse OB glomerular responses using dF/F values from each imaging frame. As a control, the vector angles were calculated from high-dimensional normally distributed random vectors. To assess whether ON–OFF angles differed from the 90° expectation for orthogonal vectors (Figures 2E and 2J), we used a one-sample Wilcoxon signed-rank test applied to *θ*_ON–OFF_-90°. This test was performed separately for locust (*n* = 11 odors) and mouse (*n* = 28 odors). One-sided tests were used when the hypothesis specifically predicted angles greater than 90°. For comparisons of ON and OFF responses to a reference odor ON response (Figures 3C and 3G), ON and OFF angle distributions were treated as independent samples, because different odors contributed separate ON or OFF values. ON and OFF angles were compared using a one sided Mann–Whitney U test to test the hypothesis that OFF-ON reference angles were systematically larger than ON-ON reference angles (mouse ON: *n* = 32 values; mouse OFF: *n* = 36 values; locust ON: *n* = 11 values; locust OFF: *n* = 12 values).

For hierarchical clustering of locust data (Figure 3D), we first calculated the summed spike counts during 4 s odor presentations for each individual PN and generated a single vector (*n* = 66 PNs) for each odor. Similarly, for mouse data (Figure 3H), dF/F values for each glomerular ROI in the mouse OB imaging datasets were averaged across the duration of an odor ON or OFF period. Dendrograms were generated using the angular distance between respective average odor responses. The cluster tree was created in such a way that the furthest pairwise distance between any two samples assigned to an individual cluster was minimized.

#### Classification analysis of on and ITI periods

To classify ON and ITI periods, we used a k-Nearest Neighbor (k-NN) classification algorithm.

For locust data, the ITI was defined as the 20 s immediately following the odor offset. The spike counts for each PN were binned in 50 ms non-overlapping time bins and averaged across the 20 s ITI period. The resulting n-dimensional PN spike count vector (where *n* = 85 PNs for Figures 5C and 5D) represented the PN activity following the odor offset for a given trial. Then the PN spike count vectors were concatenated across the 10 trials and 7 odors, resulting in 70 trials/vectors for classification. The angular distance of the resulting high-dimensional PN spike count matrix was used as a distance measure and the most represented odorant among the 10 nearest neighbors was the label assigned to each trial (i.e., 10-nearest neighbor approach; leave-one-trial-out-validation).

For mouse data, the ITI was defined as the 50 s immediately following the odor offset. The dF/F values were averaged across the 50 s ITI period for each glomerulus and the resulting n-dimensional glomerular activity vector (where *n* = 318 glomeruli for Figures 6C and 6D) represented the glomerular activity following the odor offset for a given trial. Then the glomerular activity vectors were concatenated across the 10 trials and 4 odors, resulting in 40 trials/vectors for classification (leave-one-trial-out validation). The angular distance of the resulting high-dimensional glomerular activity matrix was used to classify each trial based on the most represented odorant among the 50 nearest neighbors (i.e., 50 nearest neighbors). A larger *k*-value was used compared to the *k*-value used for locust ITI classification to account for the slower calcium indicator dynamics.

#### Time bin by time bin on response and ITI classification analysis

For locust data, the ON period was the 4 s during odor delivery and the ITI was defined as the 21 s immediately following the odor offset. The spike counts for each PN were binned in 200 ms non-overlapping time bins. The resulting n-dimensional PN spike count vector (where *n* = 85 PNs for Figures 5A and 5B) represented antennal lobe ensemble activity during odor presentations and following the odor offset. PN spike count vectors were concatenated across the 7 odors and 10 trials, resulting in 7350 time bins/vectors for classification. The odor label for each time bin was determined using a k-nearest neighbor classifier (k = 10) with leave-one-trial-out validation. A correlation distance between high-dimensional vectors was used as the metric to determine the nearest neighbors. To be rigorous and conservative, for a given trial, the 10-nearest neighbors from only other trials were used to classify (leave-one-trial-out validation).

For mouse and locust data within datasets, statistical significance was assessed using a one-sided permutation test comparing observed decoding accuracy to chance performance. For each time point, *p*-values were computed from a null distribution generated by randomly permuting odor labels across trials (1,000 permutations). To control for multiple comparisons across time, *p*-values were corrected using the Benjamini - Hochberg false discovery rate (FDR) procedure (q = 0.05), which controls the expected proportion of false positives among all significant time points. For mouse data, the ITI was defined as the 50 s immediately following the odor offset. The dF/F values were averaged every 4 frames within trials in a non-overlapping fashion and the resulting n-dimensional glomerular activity vector (where *n* = 318 glomeruli for Figures 6A–6D) represented the glomerular activity following the odor offset. Then the glomerular activity vectors were concatenated across the 4 odors and 10 trials, resulting in approximately 8,000 time-bins/vectors for classification. Again, the odor label for each time bin was determined using a k-nearest neighbor classifier (k = 10) with leave-one-trial-out validation. A correlation distance between high-dimensional glomerular vectors was used as the metric to determine the nearest neighbors. For a given trial, the 10-nearest neighbors only from other trials were used to classify (leave-one-trial-out validation).

For the analysis of OB dataset that included random presentation of odorants (Figure 6H), the ITI was defined as the 17 s immediately following the odor offset. The dF/F values for each glomerulus were averaged every 4 frames in a non-overlapping fashion and the resulting n-dimensional glomerular activity vector (where *n* = 286 glomeruli for Figure 6H) represented the temporal activity following the odor offset. Then the glomerular activity vectors were concatenated across the 11 odors and first 10 trials for each odor, resulting in 7480 time-bins/vectors for classification. Ten nearest neighbor classification analysis with leave-one-trial-out validation was performed to assign class labels to each glomerular activity vector. *p*-values were computed from 1,000 label-shuffled permutations and corrected for multiple comparisons using the FDR method described above. Time points exceeding corrected significance thresholds are indicated in the figure. Across odor accuracies were then calculated for each session (Figure 6I). Decoding accuracy was binned across five consecutive time points and compared to chance (9.1%) at each bin using one-sample t-tests (one-sided). *p*-values were corrected for multiple comparisons across time using the Benjamini - Hochberg false discovery rate procedure (q = 0.05). Time points exceeding corrected significance thresholds are indicated in the figure.
